# Draft Genome Sequence of “*Candidatus* Liberibacter asiaticus” from California

**DOI:** 10.1128/genomeA.00999-14

**Published:** 2014-10-02

**Authors:** Z. Zheng, X. Deng, J. Chen

**Affiliations:** a Department of Plant Pathology, South China Agricultural University, Guangzhou, Guangdong, China; b U.S. Department of Agriculture, Agricultural Research Service, San Joaquín Valley Agricultural Sciences Center, Parlier, California, USA

## Abstract

We report here the draft genome sequence of “*Candidatus* Liberibacter asiaticus” strain HHCA, collected from a lemon tree in California. The HHCA strain has a genome size of 1,150,620 bp, 36.5% G+C content, 1,119 predicted open reading frames, and 51 RNA genes.

## GENOME ANNOUNCEMENT

“*Candidatus* Liberibacter asiaticus” is an unculturable, phloem-limited, alphaproteobacterium associated with citrus Huanglongbing (HLB), a devastating citrus disease worldwide (1, 2). Although HLB was observed in southern China in the 1890s (3), the disease was not found in Florida until 2005 (4). Since then, “*Ca.* L. asiaticus” has been reported in several southern states of the United States. In 2012, “*Ca.* L. asiaticus” was detected in a single lemon tree in Hacienda Heights of Los Angeles County, California (5). The infected tree was quickly removed, and no other “*Ca.* L. asiaticus”-infected citrus trees have been found in California. A recent study showed that the California strain was similar to strains in the Asiatic group (6).

For the benefit of HLB research and control, this study sequenced the whole genome of the California strain of “*Ca.* L. asiaticus,” designated strain HHCA. The procedure of Zheng, Deng, and Chen (7) was followed, except for omitting the periwinkle enrichment step. DNA extract of strain HHCA was provided by Plant Pest Diagnostics Center, California Department of Food and Agriculture, with the estimate of 175 pg of “*Ca.* L. asiaticus” DNA per µl. Bacterial DNA was enriched using the NEBNext microbiome DNA enrichment kit (New England BioLabs, Inc., Ipswich, MA) and enlarged through multiple displacement amplification using the REPLI-g minikit (Qiagen, Inc., Valencia, CA). Genome sequencing was carried out by two runs of Illumina MiSeq (Illumina, Inc., San Diego, CA). The first run generated 4.4 × 10^7^ reads (mean, 242 bp), and the second run generated 3.4 × 10^7^ reads (mean, 251 bp). The whole-genome sequences of strains psy62 (8), gxpsy (9), and A4 (7) were used as references to extract “*Ca.* L. asiaticus” reads using standalone BLAST software (10) and Perl scripts. A total of 48,362 “*Ca.* L. asiaticus” reads (mean, 243 bp) were identified. With the help of Velvet 1.2.10 (https://www.ebi.ac.uk/~zerbino/velvet/) (11) and CLC Genomics Workbench 7.0 software, 239 contigs (512 bp to 39,757 bp, with ~10× coverage) were obtained. The draft HHCA genome comprises 1,150,620 bp, with a G+C content of 36.5%. Annotation was performed by the RAST server (http://rast.nmpdr.org/) (12). The HHCA genome was predicted to have 1,119 open reading frames and 51 RNA genes, and similarities are 94% to the Psy62 genome (8), 91% to the gxpsy genome (9), and 95% to the A4 genome (7).

Low “*Ca.* L. asiaticus” titers in the infected plant presented a significant challenge for sequencing the strain HHCA genome. This study performed two Illumina runs to improve sequence coverage. The first run had a coverage of ~68%, and the second run reached ~93%. More runs will further increase coverage and yet increase the sequencing cost. The draft genome sequence presented here is sufficient for the characterization of strain HHCA and will serve as a reference for studying HLB in California.

### Nucleotide sequence accession numbers.

This whole-genome shotgun project has been deposited at DDBJ/EMBL/GenBank under the accession no. JMIL00000000. The version described in this paper is version JMIL02000000.

## References

[1] Bové JM 2006 Huanglongbing: a destructive, newly-emerging, century-old disease of citrus. J. Plant Pathol. 88:37–3714 http://sipav.org/main/jpp/index.php/jpp/article/view/828

[2] Jagoueix S Bové JM Garnier M 1994 The phloem-limited bacterium of greening disease of citrus is a member of the alpha subdivision of the proteobacteria. Int. J. Syst. Bacteriol. 44:379–386. 10.1099/00207713-44-3-379 7520729

[3] Lin K-H 1956 Observations on yellow shoot of citrus. Acta Phytopathol. Sinica 2:1–11

[4] Halbert SE 2005 The discovery of Huanglongbing in Florida; presentation H3. Proceedings of 2nd International Citrus Canker and Huanglongbing Research Workshop, 7 to 11 November 2005, Orlando, FL

[5] Kumagai LB LeVesque CS Blomquist CL Madishetty K Guo Y Woods PW Rooney-Latham S Rascoe J Gallindo T Schnabel D Polek M 2013 First report of “*Candidatus* Liberibacter asiaticus” associated with citrus Huanglongbing in California. Plant Dis. 97:283. 10.1094/PDIS-09-12-0845-PDN 30722341

[6] Deng X Lopes S Wang X Sun X Jones D Irey M Civerolo E Chen J 2014 Characterization of “*Candidatus* Liberibacter asiaticus” populations by double-locus analyses. Curr. Microbiol. 69:554–560. 10.1007/s00284-014-0621-9 24912994

[7] Zheng Z Deng X Chen J 2014 Whole-genome sequence of “*Candidatus* Liberibacter asiaticus” from Guangdong, China. Genome Announc. 2(2):e00273-14. 10.1128/genomeA.00273-14 24723715 PMC3983304

[8] Duan Y Zhou L Hall DG Li W Doddapaneni H Lin H Liu L Vahling CM Gabriel DW Williams KP Dickerman A Sun Y Gottwald T 2009 Complete genome sequence of citrus Huanglongbing bacterium, “*Candidatus* Liberibacter asiaticus” obtained through metagenomics. Mol. Plant Microbe Interact. 22:1011–1020. 10.1094/MPMI-22-8-1011 19589076

[9] Lin H Han CS Liu B Lou B Bai X Deng C Civerolo EL Gupta G 2013 Complete genome sequence of a Chinese strain of “*Candidatus* Liberibacter asiaticus.” Genome Announc. 1(2):e00184-13. 10.1128/genomeA.00184-13 23640196 PMC3642251

[10] Camacho C Coulouris G Avagyan V Ma N Papadopoulos J Bealer K Madden TL 2009 BLAST+: architecture and applications. BMC Bioinformatics 10:421. 10.1186/1471-2105-10-421 20003500 PMC2803857

[11] Zerbino DR Birney E 2008 Velvet: algorithms for *de novo* short read assembly using de Bruijn graphs. Genome Res. 18:821–829. 10.1101/gr.074492.107 18349386 PMC2336801

[12] Aziz RK Bartels D Best AA DeJongh M Disz T Edwards RA Formsma K Gerdes S Glass EM Kubal M Meyer F Olsen GJ Olson R Osterman AL Overbeek RA McNeil LK Paarmann D Paczian T Parrello B Pusch GD Reich C Stevens R Vassieva O Vonstein V Wilke A Zagnitko O 2008 The RAST server: Rapid Annotations using Subsystems Technology. BMC Genomics 9:75. 10.1186/1471-2164-9-75 18261238 PMC2265698

